# Perspectives on Health Data Sharing Among Patients With Somatic and Mental Health Diseases: Focus Group Study

**DOI:** 10.2196/79990

**Published:** 2026-04-13

**Authors:** Sabrina Fesl, Caroline Lang, Falk Gerrik Verhees, Jochen Schmitt, Madlen Scheibe

**Affiliations:** 1 Center for Evidence-Based Healthcare Medical Faculty and University Hospital Carl Gustav Carus TUD Dresden University of Technology Dresden Germany; 2 Department of Psychiatry and Psychotherapy University Hospital Carl Gustav Carus TUD Dresden University of Technology Dresden Germany

**Keywords:** consent form, data sharing, electronic health records, focus groups, mental health, patient participation, qualitative research, routinely collected health data

## Abstract

**Background:**

The German Health Data Utilization Act and the Digital Act aim to enhance health data sharing for health care and research in Germany and beyond while ensuring robust data protection. A key prerequisite is patients’ willingness to share their data for primary use (PU), such as medical care, and secondary use (SU), such as research. There is a lack of qualitative research examining patients’ perspectives on data sharing under the new legal framework, especially among vulnerable groups, such as those with mental health diseases.

**Objective:**

This study qualitatively examines the factors influencing German patients’ willingness to share their digital health data for PU and SU, exploring similarities and differences between patients with somatic and mental health diseases.

**Methods:**

In 2024, we conducted 2 focus groups (FGs) with 13 outpatients: 7 with somatic diseases (FG1) and 6 with mental health diseases (FG2). Participants were recruited from a University Hospital in Dresden, Germany, based on predefined criteria. Discussions followed a topic guide with open-ended questions informed by an overview of reviews and pretests. Data were analyzed independently by 2 researchers using Kuckartz’s approach. Findings are reported according to the COREQ (Consolidated Criteria for Reporting Qualitative Research) checklist.

**Results:**

A total of 10 main categories with 32 subcategories were identified as influencing factors: previous data-sharing experience, individual usefulness for medical care, public benefit, personal and privacy concerns, data security concerns, consent management preferences, technical safety measures, legal and ethical framework conditions and requirements, informational self-determination, and social involvement and influence. Both FGs highlighted individual usefulness and public benefit despite various personal experiences. Concerns about discrimination, stigmatization, and automatic data sharing were more relevant in FG2. Technical safety measures of anonymization and pseudonymization were discussed in detail in FG1, whereas FG2 debated data protection intensively. There were concerns that data protection in Germany could potentially pose a greater health risk than the sharing of personal health data. The category consent management preferences yielded the most statements, but no clear consensus emerged. Social influence and involvement, including family, peers, and health care professionals, were more relevant in FG2. Both FGs explicitly opposed the use of health data by companies such as Google.

**Conclusions:**

This study qualitatively compared the perspectives of patients with somatic and mental health diseases. While it revealed similarities, patients with mental health diseases viewed their data as highly sensitive due to experiences of stigmatization and fear of misuse, emphasizing the need for tailored consent management. Involving family, peers, and health care professionals can increase acceptance. Health care professionals and targeted outreach can ensure transparency, raising awareness about data sharing policies to build trust, especially when commercial interests are involved. Knowledge deficits, even among tech-savvy patients, indicate the need for broad and understandable public relations efforts.

## Introduction

### Background

The German Health Data Utilization Act (Gesundheitsdatennutzungsgsetz [GDNG]) and the Act to Accelerate the Digitization of the Healthcare System (Digital-Gesetz; Digital Act), both enacted in March 2024, have created new legal regulations for using and sharing personal health data in the German health care system. The former is primarily intended to enable the use of health data for research and to facilitate its usability for purposes oriented toward the common good [1]. The latter includes the introduction of the electronic patient file. Thus, it explicitly promotes improved digital data exchange in the German health care system [2] and promises to integrate clinical data of patients across different health care institutions and throughout their lives [3]. The GDNG and the introduction of the electronic patient file as an opt-out solution are exemplary innovations that are not only relevant for health care providers and researchers but also bring opportunities and challenges for patients. In particular, the new measures aim to improve the overall quality of health care by ensuring data access for health care and research in accordance with the respective legal regulations for primary use (PU) and secondary use (SU) [4-7].

Personal health data refers to an individual’s health status and information that are uniquely identifiable to them, such as diagnoses or medication plans. It is collected during care delivery in the health care system and can be obtained from existing sources, such as electronic health records [8]. The PU of health data refers to its application to directly provide medical care to patients, such as by sharing personal health data with health care providers [9,10]. In contrast, the SU of health data extends beyond the primary goal of individual treatment. It involves the use of pseudonymized or anonymized health data for purposes other than direct patient care, including activities such as commercial and noncommercial research, public health monitoring, and developing health care policies [9,10].

Germany is pursuing a deliberate, data protection–focused approach compared with other countries’ more centralized or incentive-based health care systems. Unlike the first national centralized health data access bodies in France in 2019 (Health Data Hub) and in Finland in 2020 (Findata), Germany has launched decentralized initiatives, such as the German Medical Informatics Initiative (Medizininformatik-Initiative) in 2016 and the German Health Data Lab (Forschungsdatenzentrum Gesundheit) in 2020. The latter started to process the first requests for data use in 2025. These initiatives aim to link clinical data and medical research data to promote medical research and thus improve health care for all [11-13].

As in other European countries, patients in Germany value information, transparency, consent, and control regarding data sharing. However, Germany’s decentralized system and national federal laws such as the GDNG can make it challenging to meet these expectations consistently in all regions [14,15]. Additionally, these different framework conditions and strict German requirements for data exchange can be a challenge for the research community because there is no single, straightforward opt-out regime for all secondary research use. As a result, this can hamper collaborative research efforts, slowing progress in areas such as disease detection and treatment optimization, and reducing data usability. Overall, these challenges can lead to suboptimal individualized medical care [16,17].

Patients’ willingness to provide their health data for PU and SU is a highly topical issue for science, research, and public health care [14,18-21]. Data sharing can improve personalized care through closer cooperation between treating health care professionals and thus contribute to optimizing diagnostic and therapeutic outcomes [18]. Patients benefit from reduced treatment delays and medical errors, as well as more effective, efficient, and personalized care through access to comprehensive patient data [18,19].

In recent years, qualitative research has explored willingness to share data among vulnerable patient groups, such as patients with mental health diseases, to determine their needs, preferences, and concerns, which may differ from those of patients without mental health diseases [22-28]. These differences may require tailored approaches to promoting the responsible sharing of personal health data. While patients with mental health diseases may face stigmatization in society and in health care, which can lead to mistrust of health care providers and make comprehensive data sharing difficult, this stigmatization is less evident for patients with somatic diseases [22,25,28-30]. Compared with other patient groups, patients with mental health diseases may feel that their data are more sensitive and could be misused to their detriment or for discrimination. In contrast, patients with somatic diseases may feel that their data are less personal and more directly linked to physical health outcomes [22,23,28,31,32].

Based on the outlined new regulatory structures in Germany for digital progress, it is important to include the perspectives of patients from different target groups. Recent surveys have shown that both patients with somatic and mental health diseases in Germany are cautious and reserved about data protection [33-35]. However, the regulation of opt-out, which requires active objection, is particularly relevant for patients with mental health diseases due to the above-described potentially different concerns regarding health data sharing. Deeper insights into their attitudes are therefore crucial.

To our knowledge, there are currently no qualitative studies examining and comparing the perspectives of German patients with somatic and mental health diseases regarding sharing their personal health data for PU and SU, following the enactment of the new legal framework mentioned above.

### Objective and Research Questions

This study reports a qualitative study comparing German patients with somatic and mental health diseases who were asked about their attitudes, preferences, and needs regarding their willingness to share their personal health data for PU and SU in 2 focus groups (FGs). It shows the similarities and differences in the perspectives of both groups of patients receiving outpatient treatment in Germany, considering the context of the new regulations.

The research questions of this study were (1) What factors influence German patients’ willingness to share their digital health data for PU and SU? (2) What similarities and differences exist in data sharing perspectives between German patients with somatic and mental health diseases?

## Methods

This study was conducted as part of the PATH (Personal Mastery of Health and Wellness Data) project in January 2024.

### Study Design

Following our overview of reviews on factors influencing patients’ willingness to share their digital health data for PU and SU [9], we conducted 2 FGs with German patients with somatic and mental health diseases. FGs are useful when individual motivations and perspectives need to be explored in depth [36]. Unlike standardized interviews or questionnaires, FGs are based on a topic guide with open-ended guiding questions. Participants are free to answer according to their subjective relevance, which is also a major advantage over standardized survey methods. The moderator also has the opportunity to follow up on individual aspects to explore them in more depth. In addition, the use of a topic guide allows the discussion to be steered thematically to maintain the focus of the discussion. Therefore, FGs are particularly suitable for gaining practical knowledge for implementing data sharing concepts.

The methods and results are reported according to the COREQ (Consolidated Criteria for Reporting Qualitative Research) checklist (Multimedia Appendix 1) [37].

### Recruitment of Study Participants

Patients were recruited and enrolled at the University Hospital Dresden, Germany. To identify and recruit potential patients, physicians working in the outpatient departments of diabetology, neurology, dermatology, and psychiatry at the University Hospital Dresden were contacted in November 2023 by members of the research team in person and by email, explaining the project and planned details of the study.

Participants were selected from different clinical disciplines to obtain the most heterogeneous sample possible. Two FGs were formed, 1 with patients with somatic diseases (FG1) and another with patients with mental health diseases (FG2). Patients in both FGs were recruited by face-to-face and email with the support of project partners and staff in the respective outpatient clinics during their regular medical consultations. For this purpose, information flyers, a brief study information document, and a consent form were prepared for potential participants, containing the key details of the study (location with map, date, time, planned schedule, amount of compensation, and contact details for further questions). Participants for both FGs were recruited based on a predefined, purposive sampling plan and predefined criteria (Textbox 1).

Patients interested in participating were screened for eligibility, provided detailed information to establish a relationship prior to study commencement, and invited to attend the relevant FG by email. Consent forms were explained, and consent was obtained before the FG. Participants who attended the FG were offered financial compensation of €50 (a currency exchange rate of €1=US $1.16 is applicable).

**Inclusion criteria for participant recruitment of both focus groups.**

**Inclusion criteria**

- Aged 18 years or older
- Daily smartphone and/or tablet use
- Capable and consenting
- Able to communicate in German to enable active participation in focus group (FG) discussions

**FG1**

- Patients with somatic diseases but without a history of mental health disease (self-reported or, where possible, diagnosed through screening results or existing diagnoses) who were treated in the respective outpatient clinics during the recruitment period.

**FG2**

- Currently euthymic or at least partially remitted patients with mild, moderate, or recurrent forms of mental health disease who were treated in the Psychiatric Outpatient Clinic at the University Hospital Dresden during the recruitment period.

### Topic Guide

**Figure 1:**
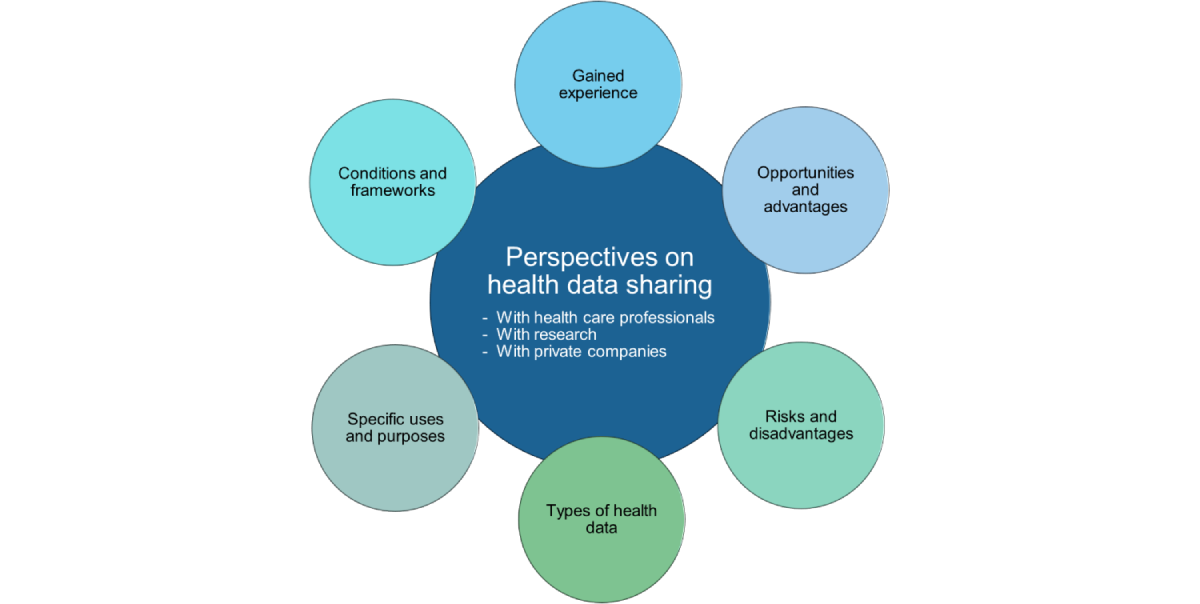
Topic selection and asked scenarios for both focus group discussions.

The topic guide for the FGs was developed based on our overview of reviews [9] on patients’ attitudes toward health data sharing for PU and SU. Guiding open-ended questions and topics were used in both FGs to allow comparisons (Multimedia Appendix 2). The list of topics (Figure 1) was presented as a word cloud on Microsoft PowerPoint slides to stimulate discussion 3 times in each FG: for health data sharing with health care professionals (PU), with research (SU), and with private companies (SU). The participants could choose 1 or more topics according to their own priorities, focus, and interests.

### Cognitive Pretests

Cognitive pretests were used to test and adapt the interview guide to ensure that all questions and items are understood and interpreted as intended and that no relevant aspects were missing. They also assessed whether the time required and the number of questions were acceptable [38]. The cognitive pretests were performed in October 2023 as individual online interviews with 7 participants with different diseases according to the predefined inclusion criteria (Textbox 1). The duration of the interviews ranged from 36 to 58 minutes. The pretests provided relevant insights and comments concerning the formulation of questions, comprehension of terms (eg, types of data), and conduct of the FGs.

### Procedure

The FGs were held on 2 evenings in January 2024 in an intimate setting of a reserved room at the University Hospital Dresden. Both were audio recorded. Three research associates with experience in qualitative research took minutes (SF, MSc; female, and MS, Dr; female, for FG1; and SF and CL, Dr; female, for FG2). Another research associate and experienced moderator (Dr; female), who was not involved in the project and had been briefed in advance, used the topic guide to lead the participants through both discussions. A resident psychiatrist (FGV, Dr; male) also took part in FG2 (patients with mental health diseases) to ensure that participants felt confident. One patient’s relative was present in each FG due to private circumstances that made this necessary at short notice. All participants agreed that the relatives could remain present; however, they did not participate in the discussion. A short presentation was provided at the beginning of each session to introduce the project goal and the researchers who were present. However, no aspects relevant to the discussion were raised to avoid influencing the participants. At the end of the discussion, the participants were given a short sociodemographic questionnaire and a validated questionnaire [39] to determine their technology commitment score.

### Data Analysis

The content of the FG discussions was transcribed verbatim, checked for accuracy, adjusted according to the rules of transcription [40], and all personal or personally identifiable information was removed. The data were analyzed using MAXQDA software (version 2018; Verbi Software GmbH) and qualitative content analysis according to Kuckartz and Rädiker’s [41] approach, in which the data are coded and evaluated according to content and thematic aspects in more detail than with Mayring’s [42] strongly theory-driven approach. This approach also enables the combination of deductive and inductive category development.

First, a category system was deductively developed based on the discussion guide and our overview of reviews [9], which was applied as a frame to the transcribed material. The category system was modified and refined as the material was reviewed in more detail, as well as through the subsequent inductive formation of recurring major categories into additional categories. Two research team members (SF and CL) independently coded the material, continuously compared emerging themes and codes, discussed data saturation through an iterative process of data collection and analysis, and checked intercoder reliability. With the help of added quotes as anchor examples, the coders could optimally match participants’ responses to the defined categories.

**Figure 2:**
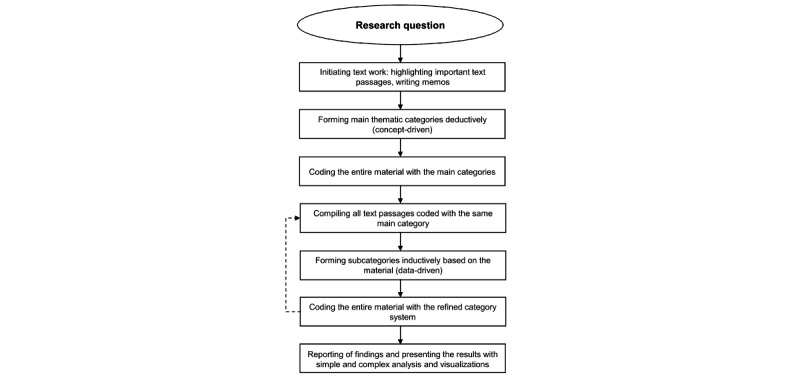
Flowchart showing each step of the data analysis process and the sequence of activities (own illustration based on Kuckartz and Rädiker [41]).

Data saturation was reached when additional data no longer produced new categories or insights, indicating that the breadth and depth of relevant information were adequately captured. To confirm saturation, MAXQDA visualization tools were used to track the frequency and recurrence of codings across datasets. Furthermore, peer discussions were conducted to validate that no significant new information appeared in the later stages of coding. This approach ensured that the coding scheme was comprehensive and that the study findings were robust and grounded in the data. This transparent and systematic approach to assessing data saturation strengthened the credibility and trustworthiness of the qualitative findings. Moreover, the research team collaboratively developed and continually refined the category system through iterative discussions to ensure that participants’ diverse perspectives were accurately represented. Figure 2 illustrates the data analysis process and the sequence of activities.

A codebook with definitions, respective anchor examples, and the number and context of statements used for qualitative coding and analysis in MAXQDA is provided in Multimedia Appendix 3.

### Ethical Considerations

The study was approved on November 6, 2023, by the Ethics Committee of Technische Universität Dresden (reference BO-EK-310072023). All procedures were in accordance with the ethical standards of the institutional ethics committee and with the Declaration of Helsinki (2024 revision). Persons with mental health conditions, considered a vulnerable population, were included in accordance with the 2024 Declaration of Helsinki (paragraphs 19-20). Additional safeguards were implemented, including assessment of decision-making capacity prior to informed consent and a resident psychiatrist (FGV) acting as a supporting person where needed, as approved by the ethics committee. Participants were informed about the purpose of the study, the voluntary nature of participation, and their right to withdraw at any time without penalty. Participants received compensation of €50 for their time and travel expenses related to attending the FG discussions. This incentive was preapproved by the ethics committee. Written informed consent was obtained from all participants who attended the FG discussions. Confidentiality was assured in compliance with the EU-GDPR (EU General Data Protection Regulation). Only researchers involved in the data analysis had access to the pseudonymized data, which were stored securely on institution-protected servers and will be deleted 10 years after study completion. Pseudonyms were used in the transcripts to protect participants’ identities.

## Results

### Sample Characteristics

#### Sample Sizes and Diseases

FG1 comprised 7 participants, of whom 3 had type 2 diabetes, 2 had a neurological disease, and 2 had a dermatological disease. The discussion lasted approximately 82 minutes. FG2 comprised 6 participants, of whom 3 had bipolar disorder, 2 had paranoid schizophrenia, and 1 had schizoaffective disorder (all currently in remission or with residual symptoms). The discussion lasted approximately 78 minutes. Because recruitment took place as part of regular daily care, it was not possible to provide a figure on refusals. A total of 2 patients dropped out on the day of the event due to acute illness.

#### Sociodemographic Characteristics

All participants’ sociodemographic characteristics (Multimedia Appendix 4) were extracted from the questionnaire provided. Regarding education, most had completed an apprenticeship (7/13, 54%). Regarding residence, most lived in large cities (9/13, 69%), followed by a medium-sized city (3/13, 23%). Regarding income, most participants in FG2 (4/13, 67%) had a monthly household income of <€1000-€1500, whereas most participants in FG1 (5/13, 71%) had a monthly household income of €3001-€3500 or €4001-€5000.

#### Technology Commitment

The total scores for the self-rated technology commitment scale and its subdomains of technology acceptance, technology competence beliefs, and technology control beliefs were high. Out of a maximum possible score of 60, the mean total score was 50 (range 41-57) for patients with somatic diseases and 45 (range 38-56) for patients with mental health diseases. The scores for each participant and FG are shown in Multimedia Appendix 5.

### Categories

#### Overview

We identified 10 overarching main categories (I-X) with 32 corresponding subcategories (Table 1). These main categories were defined to capture overarching themes relevant to the research question, whereas the 32 subcategories were developed to reflect more specific aspects and nuances within these broad themes. This approach allowed for a detailed and systematic structuring of the data, enabling the identification of patterns, differences, and relationships more precisely. The hierarchical organization enhanced the clarity and transparency of the analysis, facilitating an in-depth understanding of the participants’ perspectives. The categories in the Results section and in Table 1 were sorted according to the topic guide and participants’ response behavior. The complete codebook with definitions, respective anchor examples, and the number and context of statements used for qualitative coding and analysis in MAXQDA is provided in Multimedia Appendix 3. Each main category included in the final category system was discussed in both FGs, but with different relevance in each FG and emphasis by individual participants. Table 1 presents an overview on the number of statements per main category and subcategory in both FGs. While most statements were about consent management preferences, few were related to social involvement and influence, with only 1 comment in FG1.

**Table 1:** Main categories and corresponding first-level subcategories with total numbers of statements and number of statements per main category and subcategory in both focus groups (FGs).

Main categories and corresponding subcategories		Total statements, n	FG1 statements, n	FG2 statements, n
**Previous experience with data sharing**		38	17	21
	Health care professionals	18	10	8
	Research and clinical studies	8	5	3
	Employers and private companies	5	0	5
	Health insurance companies	4	2	2
	Medical education	3	0	3
**Individual usefulness for medical care**		32	16	16
	Comprehensive documentation	12	5	7
	Personal informedness	6	4	2
	Emergency assistance	5	3	2
	Simplification of processes	5	3	2
	Time saving	4	1	3
**Public benefit**		28	15	13
	Medical care	25	12	13
	Cost saving	3	3	0
**Personal and privacy concerns**		34	15	19
	Concerns about commercial interests	15	6	9
	Concerns being discriminated or stigmatized	8	3	5
	Personal uncertainties and skepticism	7	3	4
	Concerns being overstrained	4	3	1
**Data security concerns**		34	18	16
	Concerns about data access	24	11	13
	Concerns about data processing	10	7	3
**Consent management preferences**		128	62	66
	Consent scope	87	41	46
	Consent model	41	21	20
**Technical safety measures**		37	27	10
	Anonymization and pseudonymization	22	17	5
	Central data repository	8	6	2
	Data encryption	7	4	3
**Legal and ethical framework conditions**		35	24	11
	Data protection	11	3	8
	Standardization and regulation	10	10	0
	Ethical justifiability	7	4	3
	Monitoring and authorization	7	7	0
**Informational self-determination**		33	11	22
	Transparency	18	7	11
	Autonomy and control	8	1	7
	Trust	7	3	4
**Social involvement and influence**		9	1	8
	Family and peers	5	1	4
	Health care professionals	4	0	4

#### Key Findings

In both FGs, all participants expressed widely varying personal experiences with health data sharing, mainly regarding PU. In contrast, only those in FG2 discussed their personal experiences with employers and private companies. Regarding PU and individual usefulness for medical care, the benefit of comprehensive documentation was considered most relevant in both FGs. Regarding SU and public benefit for medical care, the advancement of drug and product development and the commercial interests of health data sharing were discussed extensively in FG2. Fears of discrimination and stigmatization were more prevalent in FG2. These fears were particularly related to disadvantages or dismissal in the workplace. Mental health diagnoses were also identified as highly sensitive in FG1. In addition, data security concerns, especially misuse, were raised more frequently in FG2 in response to the question about automatic data sharing.

It was not possible to clearly delineate participants’ views on the consent model. However, it became evident that participants in FG1 expressed only a few and rather general conditions regarding their acceptance of an opt-out model, whereas participants in FG2 attached very strict and more differentiated conditions to their acceptance of an opt-out model. University research institutions were sometimes more trusted than private and pharmaceutical research institutions, and the intended use or research project was crucial for consent preference. Participants in both FGs explicitly opposed the use of health data by “big players” such as Amazon, Google, and Apple.

Overall, technical safety measures of anonymization, or at least pseudonymization, in terms of health data sharing for SU were debated more intensively in FG1. Regarding legal and ethical framework conditions, the aspect of data protection was discussed more broadly in FG2. Aspects of standardization, regulation, monitoring, and authorization were addressed only in FG1. As facilitating factors, autonomy and control appeared more relevant in FG2, both in the context of PU and SU. Social influence, involving family, peers, and health care professionals, became much more important for participants in FG2.

Below, the results for each main category are described, with additional details and representative quotes for the corresponding subcategories from both FGs. At the end of the Results section, an overview of the key points regarding patterns and relationships between influencing factors and sociodemographic characteristics is provided.

#### Main Category I: Previous Experience With Data Sharing

Within this main category, participants distinguished between PU and SU experiences. While most reported positive experiences in terms of PU, 2 participants in each group indicated that there were bureaucratic barriers to creating an electronic health record with their health insurance companies and that the exchange of health data with their health care professionals was still paper based. Concerning SU, experiences of participating in clinical trials were more common among participants in FG2 (Textbox 2).

**Exemplary quotes on health data sharing experiences.**

**Subcategory: health care professionals**

- *So far, no doctor has been able to install anything for me; they’re all not ready yet.* [FG1.1]
- *I’ve already done this myself, [...] I’ve already allowed some practitioners, my doctor, for example, to communicate.* [FG2.2]

**Subcategory: research and clinical studies**

- *I haven’t taken part in any studies or anything like that.* [FG1.2]
- *I’ve also taken part in a few studies here.* [FG2.1]

**Subcategory: employers and private companies**

- *[…] Well, I'm not entirely sure that the diagnostic code was on the form that the employer received.* [FG2.5]

**Subcategory: health insurance companies**

- *[...] adopting the electronic patient file, at least that’s quite complicated for me.* [FG1.7]
- *[...] my health insurance says we’re still missing three documents, please submit them later. And it will be a long time before I can even go to rehab.* [FG2.4]

**Subcategory: medical education**

- *I had just been to a lecture with one of his superiors in December.* [FG2.4]

#### Main Category II: Individual Usefulness for Medical Care

A total of 5 subcategories emerged on individual usefulness for medical care when sharing health data. If a patient changes location or physician, the medical history is fully available to the next practitioner, which could prevent undue disease progression. Benefits were also perceived for obtaining a second medical opinion and to avoiding bringing paper copies of medical reports to the appointment. In addition, rapid assistance in an emergency and saving time for administrative formalities, such as pension procedures or medical history interviews, were discussed (Textbox 3).

**Exemplary quotes on individual usefulness for medical care.**

**Subcategory: comprehensive documentation**

- *Because then it’s basically my ID that I can present when I go to a new doctor.* [FG1.5]
- *If they had everything stored on the health card and the medical service, the doctors and everyone would had access to it straight away. That would make things much easier.* [FG2.4]

**Subcategory: personal informedness**

- *[...] that the documentation has become more open. [...] whereas today you are basically presented with complete data collections and can also [...] assess them yourself.* [FG1.3]
- *I have to say, I have a folder at home that is now so thick [...] that would of course be an advantage for me if it were completely stored on the health card.* [FG2.4]

**Subcategory: emergency assistance**

- *I can’t imagine how this can be handled properly, especially in emergencies.* [FG1.7]
- *You always have to assume that if you suddenly find yourself in a situation like this, a doctor needs to know something. And you can’t say that yourself.* [FG2.1]

**Subcategory: simplification of processes**

- *Because that would always simplify and accelerate many processes.* [FG1.2]
- *And I know that the doctor will only use them in a positive way, not against me, but so that my treatment can run smoothly.* [FG2.2]

**Subcategory: time saving**

- *[...] that would also shorten the anamnesis interview, which always has to be conducted. Then you can shorten it considerably, he can ask exactly the questions he needs to ask [...] I think it would be great if this could be done automatically in advance. That would be really useful.* [FG1.2]
- *I wouldn’t need to talk about it for long; they would have it directly in the app or on the card.* [FG2.3]

#### Main Category III: Public Benefit

Regarding possible public benefits, drug and product development was discussed by 5 participants in FG2, which was linked to their gratitude that new drugs are being developed, meaning that treatment for patients with mental health diseases is improving. Regarding statistics and achieving gender-neutral, high-quality health care, there was agreement that health data should be collected automatically, especially if they are anonymized. The aspect of cost savings in health care by treating individuals well enough to enable them to remain active and able to work in the long term was raised by 1 participant in FG1 (Textbox 4).

**Exemplary quotes on public benefit.**

**Subcategory: medical care > drug and product development**

- *Because I can imagine that such a secondary gain [...] if, for example, a hoover manufacturer now has the data and sees, okay, we have an increase in respiratory diseases in the population [...]. Then we have a larger market, they are no longer so expensive [...] and people with hyper allergenic diseases would also benefit from this at some point.* [FG1.2]
- *And research depends on the results of the patients. It’s not possible otherwise. All the examinations have to be compared somehow.* [FG2.1]

**Subcategory: medical care > gaining knowledge**

- *There is also a great deal to be learned from the data collected.* [FG1.7]
- *Yes, that now, in a few years’ time, there might be another patient who the doctors have helped by using my data, for example.* [FG2.3]

**Subcategory: medical care > deriving preventive measures**

- *Or even the health insurance company, because they look at preventive measures, that’s okay too.* [FG1.6]
- *You can really use these neural networks to see how high the probability is that if the person has disease A and then has disease B, or just has the symptoms that you can then set up better drugs.* [FG2.6]

**Subcategory: cost saving**

- *And the biggest costs come from early retirement. Not the cost of medication [...] That would be an incredibly great contribution.* [FG1.6]

#### Main Category IV: Personal and Privacy Concerns

Concerns about commercial interests were present in both FGs. Skepticism about possible conclusions from anonymized health data or data sharing without prior information and the associated potential for discrimination or stigmatization was particularly expressed in FG2. Contrary to the benefits of comprehensive and transparent documentation, concerns about being overstrained by the large amount of data available were also expressed (Textbox 5).

**Exemplary quotes on personal and privacy concerns.**

**Subcategory: concerns about commercial interests**

- *But I think that at least a not inconsiderable part of the research should be made available to the general public again. Because if the general public has contributed to the data, then the general public should not be ripped off again in the end.* [FG1.4]
- *I can imagine [...] Amazon says we also want to do research [...], and then they have such an offer for us, and earn money with it. Nowadays, data is the most important thing where you can really make money and where you also earn money.* [FG2.6]

**Subcategory: concerns being discriminated or stigmatized**

- *[...] health insurance company XY now wants to consider that it no longer wants to accept expensive patients in the near future.* [FG1.6]
- *I’m just going to be open and honest, due to the severity of my disease, I’m constantly afraid that somehow things could be spread about me and that people know me [...].* [FG2.2]

**Subcategory: personal uncertainties and skepticism**

- *Of course, I also have the information paper-based, and I get what I ask for. Although in some cases I have to ask for, such as this or that medical report. But I always get what I ask for. Simply saving the paper version is of relatively little use to the next practitioner. Because there really hasn’t been enough development [in terms of digitalization]. At least not in the short time frame in which it should be in the end.* [FG1.7]
- *If my data is used for this, even though I have absolutely no idea about it, I would find that a bit creepy, yes.* [FG2.2]

**Subcategory: concerns being overstrained**

- *The disease is like this [...] I don’t want to know everything that’s bubbling and boiling inside me. I want to be able to live with the disease myself to some extent, and I push the other things aside.* [FG1.1]
- *That can also develop into a pretty big mental chaos [...], quite honestly.* [FG2.4]

#### Main Category V: Data Security Concerns

Regarding data security, participants differentiated between concerns about data access and data processing. It was particularly emphasized in FG2 that health data are generally very sensitive. The data must be protected against unauthorized access and changes and should not be used, for example, for research purposes without notification. Contrary to existing concerns about data security, there was a statement from FG1 that the high level of data protection in Germany may sometimes be riskier for health than sharing one’s own health data (Textbox 6).

**Exemplary quotes on data security concerns.**

**Subcategory: concerns about data access**

- *It gets bad when someone gets into the data collection and changes the blood group, for example.* [FG1.1]
- *So, basically, that’s the fear that everyone has. Who can access all the health data?* [FG2.1]

**Subcategory: concerns about data processing**

- *[...] when I have my orthopedist, my oncologist, my ophthalmologist [...], I can find an individual person there.* [FG1.6]
- *But people would have absolutely no idea what happens to their data. They wouldn’t even know what studies their data is being used for.* [FG2.2]

#### Main Category VI: Consent Management Preferences

##### Consent Scope

Regarding the scope of consent, a distinction was made between sensitive and nonsensitive health data. Communicable diseases, sexually transmitted diseases, mental health diseases, and health care professionals’ subjective assessments of patients in open notes, such as on their behavior during treatment, were categorized as highly sensitive and consequently associated with a restriction or rejection of sharing. As one possible approach, the creation of different categories of health data was mentioned. Health data in anonymized or pseudonymized form were also considered particularly notable, and emergency data were discussed as a special case in FG2 only (Textbox 7).

Further subcategories were developed, differentiating consent scope by recipient and purpose and dividing statements related to PU (health care professionals) and SU (research, pharmaceutical, and private companies). Regarding PU, while some participants expressed the desire for limited data sharing only and the relevance of a complete dataset for an optimal, holistic treatment, others expressed agreement with full sharing. Opinions included limited selection with prior consent to the treating physician, sharing only with relevant treating physicians, refusal of automatic sharing, and emergency situations as an exception. In FG1, health insurance companies and company physicians were discussed as special recipients for whom exceptions should be possible to avoid disadvantages at work or regarding health insurance fees. Regarding SU, the private companies mentioned included suppliers of medical devices and medical technology (Textbox 8).

**Exemplary quotes on consent scope related to the type and format of information.**

**Subcategory: sensitive vs nonsensitive data**

- *There are infectious diseases, for example. I wouldn’t know whether I would authorize everyone to see them.* [FG1.5]
- *But now I’m asking myself, even in the context of psychotherapy, private historical data is told. For example, it could be a case of abuse. Can that also be health data?* [FG2.6]

**Subcategory: anonymized and pseudonymized data**

- *But really just the anonymized collection of the data. And I would agree to that without any reservations.* [FG1.7]

**Subcategory: emergency data**

- *I could imagine that, as I said, the important things are visible to everyone. That every doctor can look at my allergies, but by no means that every doctor gets the other data […].* [FG2.6]

**Exemplary quotes on consent scope related to the recipient and purpose.**

**Subcategory: health care professionals**

- *In principle, I feel the same way about selection, as I would intuitively say that not every doctor should be able to see everything.* [FG1.4]
- *Well, on the whole, I wouldn’t care […]. If this is going to be widespread in the medical field without my consent, it might make sense to have some sort of prioritization. In other words, doctors in private practice and hospitals would be allowed to access it and not, let’s say, anyone who has anything to do with medicine.* [FG2.5]

**Subcategory: research and pharmaceuticals**

- *The only important thing is the purpose, i.e., medical research, whether it is university research or a pharmaceutical company, I wouldn’t care. [...] The main research work is done by the pharmaceutical companies.* [FG1.7]
- *I also share my data if I can decide that. [...] I think that’s something everyone should be able to decide. So, I don’t think it is right that everyone should be able to extract something from it.* [FG2.5]

**Subcategory: private companies**

- *[...] I wouldn’t make that much of a distinction between medical device manufacturers and other manufacturers.* [FG1.2]
- *I wouldn’t give my data to just any company, especially if I don’t know what they need the data for and what they want to do with it.* [FG2.2]

##### Consent Model

The consent model subcategory covers whether participants preferred an opt-in or opt-out solution, reflecting our guiding question on automatic health data sharing for PU and SU. By definition, active consent and permission to use and share health data, or explicit and flexible consent, were coded as opt-in. Standard consent with an active and explicit right to refuse the use and sharing of health data was coded as opt-out. The answers in both FGs were very heterogeneous, and no clear picture could be derived. However, it became apparent that patients in FG1 tended to mention only a few and rather general conditions for their agreement to an opt-out model, whereas patients with mental health diseases set strict and more differentiated conditions for their acceptance (Textbox 9).

**Exemplary quotes on the consent model.**

**Subcategory: opt-in**

- *Specifically, which doctor gets access? And only that doctor will be authorized.* [FG1.3]
- *And I think it’s very important that as part of the research [...] they are asked: ‘would that be something for you? Would you take part?’* [FG2.4]

**Subcategory: opt-out**

- *So, with this option, I would be able to deselect something, i.e. deny someone access. But in general, if I don't say anything, I want to allow access.* [FG1.7]
- *I could imagine a system or principle similar to that of organ donation, where you say that all data is basically free and if I don’t want it [...] then I actively disagree.* [FG2.5]

#### Main Category VII: Technical Safety Measures

Anonymization, or at least pseudonymization, serves as a facilitator of health data sharing for SU. A central data repository, such as the storage of anonymized health data on secure server platforms, preferably in Germany, or in a registry, would support controlled access for SU. Data encryption, possibly managed by entering a personal identification number, was considered relevant to the design of related software (Textbox 10).

**Exemplary quotes on technical safety measures.**

**Subcategory: anonymization and pseudonymization**

- *Another issue is anonymized use, as is the case in the Scandinavian countries. A lot of insights can be gained from the collected data.* [FG1.7]
- *And if they actually collect individual data from me [...], it would have to be designed in such a way that you can no longer recognize which patient it is.* [FG2.2]

**Subcategory: central data repository**

- *So, I would like this intermediate instance [...] the data goes to a register. And I’m sure that not just anyone can access it [...] that it doesn’t go to a company.* [FG1.6]
- *As I said, in the end it is stored somewhere on a large server, and the question is then with the image, I think that’s a good idea.* [FG2.4]

**Subcategory: data encryption**

- *I think it would be very good if this is encoded on the insurance card and can also be read by my doctor after I have given my consent.* [FG1.3]
- *Exactly, and then the rest of the data, which can perhaps also be encrypted in parts. And when I go to the doctor, the doctor shows me what he wants to open and I enter a PIN.* [FG2.6]

#### Main Category VIII: Legal and Ethical Framework Conditions and Requirements

The most frequently mentioned aspect of this main category, especially among participants in FG2, was privacy. The importance of employers not having access to health data was especially highlighted. There was a call for effective data protection under the EU-GDPR. The paradox was also described, as some individuals reveal everything on social media but insist on high data protection when sharing health data. It would be important to create a harmonized legal framework, binding regulations, and a standardized digital data recording system valid throughout Germany, but this was viewed as hardly feasible. Participants in FG1 would be more willing to share their health data for SU if the appropriate and ethical use of health data were ensured through authorization procedures and monitoring by an independent committee (Textbox 11).

**Exemplary quotes on legal and ethical framework conditions and requirements.**

**Subcategory: data protection**

- *The fact that any human resources director can then access it should be avoided.* [FG1.1]
- *And in this context, it is very important to me that my health data is very well protected and cannot be viewed by third parties, because I don’t want so many people to find out about it.* [FG2.2]

**Subcategory: standardization and regulation**

- *And such regulations and procedures are needed to get things off the ground.* [FG1.1]

**Subcategory: ethical justifiability**

- *[...] they would still have to say at the beginning what they want to research it for. This ethical component would have to be clarified.* [FG1.6]
- *They wouldn’t know at all which studies their data would be used for. And they have no way of deciding, even from an ethical point of view, whether this is good for them or not.* [FG2.2]

**Subcategory: monitoring and authorization**

- *The use of the data must, of course, also be justified and authorized by the Commission or something else to ensure that it is used sensibly.* [FG1.7]

#### Main Category IX: Informational Self-Determination

This main category included key aspects that enable individuals to make and act on their own decisions: transparency, trust, autonomy, and control. The latter was considered relevant in the context of PU and the self-determined decision of which physicians will see individual health data. In particular, participants in FG2 considered the implementation of robust control mechanisms important because of anticipated limited public trust in sharing health data. Regarding SU, autonomy should be respected so that, in principle, everyone can decide whether to share their health data for research. Participants also expressed interest in protocols for transparently reporting information to each individual about their contribution and support of research activities, possibly combined with a mandatory disclosure of research results (Textbox 12).

**Exemplary quotes on informational self-determination.**

**Subcategory: transparency**

- *And I think the minimum would be to have complete transparency about how this system works.* [FG1.4]
- *Well, I would definitely want to know if the data is being used for research. I would also not object to the data being used for research purposes in principle.* [FG2.2]

**Subcategory: autonomy and control**

- *Of course, not everyone can do that. You have to have it in your own hands to authorize it, or not.* [FG1.1]
- *I also share my data if I can decide that. So, I’m open to that. As I said, I think that’s something everyone should be able to decide.* [FG2.5]

**Subcategory: trust**

- *But to start with [...] you just have to assume a bit of trust, otherwise nothing will work.* [FG1.1]
- *Their intention is certainly to show that people trust research rather than companies [...] Whether it is Siemens or […] University.* [FG2.6]

#### Main Category X: Social Involvement and Influence

The topic of social influence was not addressed at all in FG1, apart from 1 statement on data sharing with the employer. In FG2, it was argued that family members or peers could also be involved in decision-making and that family members should also be allowed to release health information in an emergency. Health care professionals could play a supportive and consultative role, such as by providing information about data sovereignty and personal responsibility for the electronic medical record (Textbox 13).

**Exemplary quotes on social involvement and influence.**

**Subcategory: family and peers**

- *Because, for example, I had just started at a company [...] with the diagnosis [...]. And there was even the recommendation from my family members [...]: ‘Lie about it, don’t say it’.* [FG1.6]
- *[...] or you know something like a password or a very long string and you can enter it, even as the person’s husband or wife, and then also disclose the other data.* [FG2.6]

**Subcategory: health care professionals**

- *That's how it was explained to me. In principle, I am responsible for this [electronic patient] file. So, I can say that the doctor can see various things and the doctor cannot see various things.* [FG2.3]

### Patterns, Differences, and Relationships Regarding Sociodemographic Characteristics

#### Gender

The category of personal and privacy concerns was predominantly articulated by female participants. The concerns about discrimination or stigmatization expressed were concrete and related to disadvantages in professional life and social contexts. By contrast, male participants emphasized the category of informational self-determination the most, especially the points of autonomy and control. Statements such as “But that everyone can then decide whether to approve data sharing or not” (FG2.5) illustrate a desire for fundamental sovereignty and the ability to make independent decisions. Furthermore, male participants called for clearer rules and more rigorous monitoring within the legal and ethical framework conditions and requirements. Their priority was to establish a reliable, externally controlled system that could be trusted.

#### Age

For older participants (aged 49-79 years), the focus was on data security concerns and informational self-determination principles. As with male participants, autonomy and control were central to this group. The core issue was the fundamental right to control how one´s own data are used. There were general but strong concerns voiced about the overall security of the system, which often failed to address technical details. Concerns regarding data access emerged leading in this group with statements like “But the abuse that results when someone who has no actual responsibility for it gets hold of the date is the fundamental fear” (FG2.1). Conversely, younger participants (aged 25-43 years) focused on concrete use cases and the practical individual usefulness for medical care. Emergency assistance was a frequent topic of discussion, highlighting a willingness to share health data in a clearly defined context. Younger participants expressed very granular preferences regarding the consent model. Within the subcategory of recipient and purpose, they specified which health data should be visible to which health care professionals (eg, general practitioner vs specialist), indicating a desire for flexibility and adaptability within the scope of consent.

#### Socioeconomic Status

The characteristics of education and income revealed overlapping patterns in the FG discussions and are therefore analyzed together. The analysis showed that socioeconomic status strongly influences the nature of the perceived positive and negative consequences of sharing health data. Participants with higher education and/or higher income (>€3000) made arguments for or against sharing data on a more systemic level. Participants in this group discussed technical safety measures in detail, citing anonymization and pseudonymization as prerequisites for consent.

When personal and privacy concerns were raised, they primarily fell within the subcategory of concerns relating to commercial interests:

It will always result in advertising and similar things. So, I think there will be enough creative ways within the industry to use health data to reach target groups even more precisely, to market certain products even more accurately, and so on.

FG1.4

The 2 participants with the highest income, who have a university degree and are in the middle of their professional lives, explicitly supported the idea of an opt-out solution. However, they linked this to clear conditions that meet their needs for both individual usefulness and individual control.

By contrast, participants with lower and/or middle levels of education and/or lower incomes raised more specific concerns related to personal matters and privacy. As with female participants, the subcategory of concerns related to discrimination or stigmatization was extremely dominant. The fear of job loss or disadvantages with insurance was existential. Another focus was on trust: while the positive social influence of health care professionals and trust in the treating physician were cited as anchors, trust in anonymous, digital systems was low.

## Discussion

### Overview

For this study, we conducted 2 FGs and analyzed the data using qualitative thematic analysis to explore the perspectives of German patients with somatic and mental health diseases on health data sharing. We explored similarities and differences between the 2 patient groups regarding factors influencing their willingness to share health data for PU and SU. Analogous to the order of the Results presentation, the findings are now discussed in the context of the current state of research.

### Discussion of Principal Findings

#### Previous Experience With Data Sharing

The majority of participants were already experienced in sharing health data, with a predominantly positive response. While 3 participants from each FG reported some negative experiences, they were still willing to share their health data with health care professionals. This suggests that the individual usefulness for medical care can outweigh these negative experiences. In the context of PU, an interview study of the general population in Poland in 2024 showed that positive experiences and frequent use of eHealth solutions are important facilitators for health data sharing [43]. Similar findings regarding SU have been reported in the literature, where 98% of these participants considered the altruistic and public benefits of data sharing to outweigh the potential risks [44].

#### Individual Usefulness for Medical Care

Patients in both FGs were highly supportive of sharing health data with their health care providers, especially to support personalized medical care. This finding is consistent with previous qualitative studies [27,45,46] postulating benefits for better treatment through comprehensive documentation, such as avoiding multiple diagnoses. However, a scoping review [47] concluded that relevant sensitive information about patients’ mental health was regularly missing from electronic health records because they follow a more narrative reporting style, negatively impacting the flow of information in the treatment process. Nonetheless, in this study 1 patient per FG argued that an electronic patient file should include only objectifiable data, such as laboratory parameters, and not subjective assessments.

These findings are also consistent with 2 qualitative studies [22,48] regarding the potential to increase personal informedness and simplify processes. Personal access to medical records can help patients with mental health diseases improve their understanding of their diagnosis, their mental health status over time, and the reasons for their medical treatment, as well as help them remember their previous visits to the physician. In addition, access to personal data increases the sense of control over one’s own health care [49]. Emergency assistance and time-saving aspects were frequent arguments for health data sharing, especially for younger participants, and also echo previous qualitative studies [43,50].

#### Public Benefit

In both FGs, the potential for improvements in medical care through the sharing of health data was seen as a key public benefit. This was considered in 2 ways: in terms of drug and product development and in terms of gaining knowledge, for example, for evidence-based public policy decision-making and deriving preventive measures from health statistics. A current study using German survey data and a survey experimental vignette design confirmed that individuals understand how valuable their data are for researchers in terms of SU and possible public benefits [51].

Participants in FG2 particularly supported the work of the pharmaceutical industry and acknowledged the improvements that have been achieved over the years through the development of drugs for the health care of patients with mental health diseases. An interview study involving patients with mental health diseases in the United Kingdom revealed a desire to alleviate their suffering, as well as the expectation of personalized medicine, tailored treatments, and improved disease management in the population [28].

#### Personal and Privacy Concerns

In this study, especially participants with higher levels of education and/or higher incomes had major concerns about commercial interests, particularly regarding the use of health data by technology companies, which were discussed extensively by FG2. These findings are consistent with the existing literature of other FGs and an online survey in the United Kingdom [46,52,53]. Similarly, concerns about discrimination and stigmatization were more prevalent among the FG2 sample, particularly among participants with lower and middle levels of education and/or lower incomes, and among women. These fears, particularly discrimination by insurance companies and employers in the context of SU, have been previously reported [28]. In a qualitative study, mental health service users expressed concerns about institutional discrimination [52]. The stigma associated with mental health diseases has hindered patients’ acceptance of electronic communication and data sharing, and limited the broader use of this technology among underserved populations [29], making the reduction of stigmatization particularly important. Moreover, participants in both FGs also expressed concerns about being overstrained by the amount of accessible documentation and the wide availability of data, findings that are consistent with those of a qualitative study in Belgium involving vulnerable patients [22].

#### Consent Management Preferences

Overall, the results of this study show that no clear consensus has emerged regarding preferences for consent management, but fundamental differences were found between the characteristics of diseases and sociodemographic data. The aspects described below may explain our inconsistent findings and highlight the importance of granular personal control over data access, limited datasets, restricting specific information, purposes and recipients, and choices regarding health data sharing. A qualitative study exploring the public’s hopes and fears about health data sharing has shown that these perspectives are strongly influenced by an individual’s personal circumstances [54].

Regarding PU, some patients in both FGs expressed a desire for limited data sharing, while others agreed to full sharing of data. Younger participants expressed very specific preferences regarding which health care professionals should have access to which health data (eg, general practitioner vs specialist). Among other things, mental health diseases were categorized as highly sensitive in both FGs, and consequently associated with restrictions on data sharing or even rejection. A systematic review including survey and interview data also demonstrated greater agreement to share nonsensitive digital health data, with mental health, drug or alcohol use, and sexually transmitted diseases being considered particularly sensitive [55]. Regarding SU, our results suggest that university research often received greater support than pharmaceutical research, and participants in both FGs were explicitly opposed to the use of health data by Amazon, Google, and Apple, although artificial intelligence could transform patient care through its applications and augmentation of medical knowledge [56]. Our results on differentiation by recipient and purpose reflect the findings from studies in Germany [35,57] and other European countries [53,58]. A recent German cross-sectional survey also showed that only about 29% of the population would share their health data with private research institutions, and 66% are unwilling to share their health data with commercial companies, especially not technology companies [45].

While 2 participants with the highest income, who have a university degree and are in the middle of their professional lives, expressed a preference for opting out with their own personal options, others felt that the ability to make fundamental, independent choices was vital. This aligns with studies indicating that greater openness to data sharing is associated with higher digital literacy and socioeconomic status when control mechanisms are in place [18,35,59]. However, concerns about the reliability of our findings must be raised, as the distinction between opt-in and opt-out may have been ambiguous. At times, it appears that opt-out was interpreted as “inconvenient,” as noted by 1 participant in FG1, and might indicate insufficient knowledge on this issue. In an Irish FG study involving 85 participants by Flaherty et al [58], opt-out was considered more convenient because it avoids the need for repeated consent for similar studies and is more cost-effective. Similarly, participants in a recent interview study in Germany emphasized the convenience and effectiveness of opt-out [57]. An interview study with health care professionals for patients with mental diseases also supports the hypothesis of a possible lack of knowledge, where most respondents (75%) felt that their patients did not fully understand the process and consent forms for data sharing [24]. The identified knowledge gap found in this study and elsewhere underscores the need for thorough public information about the different consent options and existing regulations, some of which have already been implemented. At the European level, the European Health Data Space (EHDS) regulation, in force since March 2025 and applicable from March 2027, encompasses different key milestones [60]. By 2029, each member state must have established a national health data access body [61]. Details of the EHDS are currently being elaborated in European projects (eg the Joint Action Towards the European Health Data Space 2) [62]. A workshop focused on the perspectives of different stakeholders on the opt-out procedure within the EHDS, as well as how Germany can structure the opt-out procedure and rights of objection within this framework [62,63].

#### Legal and Ethical Framework Conditions

The issues of data protection and ethical justification for data sharing were particularly noteworthy to both of our FGs. The participants in FG2 strongly emphasized that health data are generally very sensitive and need special access protections. Patients with somatic diseases, particularly male patients, requested special monitoring and authorization measures. Although data protection in Germany is already very robust and a deliberate, data protection–focused approach is followed, there appear to be evident concerns and a lack of knowledge [11]. In an FG study in the Netherlands, Wetzels et al [64] showed that patients had limited knowledge of data protection practices. These results demonstrate the need for broader and targeted public relations work to better educate the public on this issue. Germany provides information for the interesting public on websites, interactive learning platforms, and in journals, which also include information on data protection [65-70].

#### Informational Self-Determination

Especially the points of autonomy and control were fundamental for the male and older participants aged 49-79 years. These 2 aspects, as well as trust, were consequently identified as facilitators of health data sharing in both FGs and elsewhere [71,72]. A Finnish study using survey data to develop a theoretical model found that trust and control are key mediators between attitudes and willingness to share health information, with trust being more influential than control [73]. However, a questionnaire study from Denmark revealed a strong desire for control over personal health data, even when there is trust in the institutions using it [74]. In a computer-assisted telephone interview study conducted in Germany, trust emerged as the most significant factor to increase the willingness to share personal health data [59].

#### Social Involvement and Influence

Positive social influence of health care professionals and trust in the treating physician can encourage health data sharing according to 2 participants in FG2 with lower levels of education and/or lower incomes. The existing literature certainly indicates a substantial influence of physicians’ behavior on patients’ willingness to share their health data [75]. In patients with mental health diseases, physicians may underestimate the importance of physical complaints and attribute them to a mental disorder, which can lead to misdiagnosis and poorer treatment outcomes. This phenomenon of “diagnostic overshadowing” can lead to a loss of trust in the treating physician, particularly in those with mental health diseases, reducing their willingness to share their health data [52,76-78]. Positive encouragement and clear, open communication strategies from health care professionals can be important facilitators and were found to significantly influence outcomes [79]. Specifically for this purpose, a patient handout in various and simplified languages, as well as waiting room videos and posters for German medical institutions, were developed [70]. Moreover, German statutory health insurance companies provided their patients with comprehensive information materials [80], and an interactive learning platform for experts is gradually being established [68].

### Strengths and Limitations

A major strength of this study is the diversity of its cohort in terms of gender, age, and somatic and mental health diseases. The diversity of these characteristics was considered during the recruitment process, as it allows access to different perspectives from patients with different chronic and mental health diseases. In particular, the opinions of patients with mental health diseases are still rarely prioritized in research on health data sharing, even though they represent more than one-third of the population [81]. The group dynamics and observable interactions in FGs provide valuable and deeper insights than face-to-face interviews. Another strength of this study is that the FGs were able to show how participants’ views are formed and how they change through social interaction. This allowed us to better contextualize the verbal data.

However, our findings may have been biased by problems recalling experiences in the distant past. Our participants also represent a convenience sample, as participants were recruited from the University Hospital in Dresden, Germany. In addition, our cohort may have been affected by selection bias due to the financial compensation of €50. Moreover, investigated relationships between sociodemographic characteristics like age, gender, education, and income levels as possible influencing factors may only be considered of limited significance due to the small sample size. Furthermore, our recruitment of only relatively tech-savvy patients who used a smartphone or tablet daily to increase FG efficiency may have introduced selection bias, potentially resulting in higher technology acceptance scores. Besides, our results may have been influenced by volunteer bias. In addition, our results may have been influenced by social desirability, as participants may have feared being judged by other FG members if they provided truthful answers. Finally, qualitative research findings are not designed to be generalizable. However, an exploratory study design enables patient participation, as emphasized in the GDNG [1], and allows for a deeper and more comprehensive understanding of patients’ perspectives, as well as facilitating the development of hypotheses to inform future research and practice.

### Implications for Future Research and Practice

Further validation of the findings of our exploratory qualitative study on data sharing can be achieved through future research with a representative sample. This research could also reveal possible correlations with education, income, or place of residence, which have already been identified at the international level [9]. Future qualitative and quantitative research comparing the attitudes of patients with somatic and mental health diseases, as well as healthy individuals, could deepen our understanding of their unique needs and concerns. Investigating the attitudes of individuals with poor mental health could also be beneficial, despite the potential challenges of recruitment.

Social influence of family members and especially health care professionals had a much greater impact on patients with mental health diseases and for those with a lower socioeconomic status. Therefore, the latter should be involved in future research and, after the aforementioned training measures, be empowered in their role as multipliers to inform patients about the potential benefits and risks of sharing health data. Research should also assess the effectiveness of involving family members, peers, and health care professionals in decision-making processes. This research can refine governance frameworks and improve patient-centered approaches to health data sharing. It can also lead to powerful medical artificial intelligence systems, such as clinical decision support systems, that are trained on higher-quality and more representative data.

The issue of data protection was more important for patients with mental health diseases. Both they and the female participants expressed greater fears of discrimination and stigmatization. To reduce the identified knowledge deficits, which have even become apparent among tech-savvy patients, it is crucial to implement broad education and public relations initiatives that provide easy-to-understand information about the very high data protection standards and the procedure for storing and transferring health data. In addition, there needs to be greater awareness of the different levels of consent options, such as only allowing certain physicians to access individuals’ electronic health data.

### Conclusions

The use of health data for PU and SU is a valuable capability of a modern health care system and the society it serves. Therefore, patients’ and health care professionals’ perspectives must be considered when designing the regulatory framework. This study provides insights into the different perspectives and preferences of patients with somatic and mental health diseases regarding data sharing. In particular, those with mental health diseases viewed their health data as highly sensitive due to previous experiences and fears of discrimination and stigmatization. Therefore, tailored consent management and involving patients in health data sharing were highly preferred. Participatory approaches involving family members, peers, and health care professionals can also help to reduce fears and increase acceptance. Health care professionals play an important role, as they can ensure transparency through targeted educational work and actively sensitize patients to the principles of data sharing, which can be supportive, even if commercial interests are involved. The knowledge deficits identified in this study, which also existed among tech-savvy patients, indicate that broad and easily understandable awareness-raising and public relations initiatives are needed. They can help inform the population comprehensively about the very high data protection standards, the procedure for storing and transferring health data, and the personalized consent options.

## Appendix

Multimedia Appendix 1 COREQ checklist.

Multimedia Appendix 2 Guiding questions and topics for both focus groups.

Multimedia Appendix 3 Codebook with definitions, respective anchor examples and number / context of statements used for qualitative coding and analysis in MAXQDA.

Multimedia Appendix 4 Participant characteristics on sociodemographic characteristics.

Multimedia Appendix 5 Participant characteristics on technology commitment scores.

## Abbreviations

EHDS: European Health Data Space
EU-GDPR: EU General Data Protection Regulation
FG: focus group
GDNG: Gesundheitsdatennutzungsgsetz
PATH: Personal Mastery of Health and Wellness Data
PU: primary use
SU: secondary use
