# Supplementary material for: Perspectives on Health Data Sharing Among Patients With Somatic and Mental Health Diseases: Focus Group Study
Source: J Med Internet Res. 2026 Apr 13;28:e79990. doi: 10.2196/79990 (PMC13122138; doi:10.2196/79990)
Supplement: Multimedia Appendix 2 [file jmir_v28i1e79990_app2.docx]

**Icebreaker question**

*What is the first thing that comes to mind when you think about health data?*

*(Timeframe: 10-15 minutes)*

**Guiding questions and topics**

| Question 1: How do you feel about the use of your health data by your healthcare providers? |
| --- |
| Question 2: How do you feel about the use of your health data for research? |
| Question 3: How do you feel about the use of your health data by private companies? |

| Topic a: Experience in health data sharing |
| --- |
| Topic b: Opportunities or advantages of health data sharing |
| Topic c: Risks or disadvantages of health data sharing |
| Topic d: (Types of) data for sharing |
| Topic e: Specific uses or purposes for health data sharing |
| Topic f: Conditions or frameworks for health data sharing |

1. *How do you feel about the use of your health data by healthcare professionals?*
2. Do you already have experience with this?
   - - - If yes, how was your experience?
       - If no, what are possible reasons?
3. What opportunities or advantages do you see in the use of your health data by healthcare professionals?
4. What risks or disadvantages do you see in the use of your health data by healthcare professionals?
5. What (type of) health data would you share with healthcare professionals?
6. For which specific uses or purposes would you share your heath data with healthcare professionals?
7. What framework conditions must be met for you to consider health data sharing with healthcare professionals?
8. Imagine that your health data would be automatically made available to healthcare professionals if you do not actively opt out. How does it feel?

*(Timeframe: 20 minutes)*

1. *How do you feel about the use of your health data by research companies?*
   1. Do you already have experience with this?
      - - If yes, how was your experience?
        - If no, what are possible reasons?
   2. What opportunities or advantages do you see in the use of your health data for research?
   3. What risks or disadvantages do you see in the use of your health data by research companies?
   4. What (type of) health data would you share for research?
   5. For which specific uses or purposes would you share your heath data with research companies?
   6. What framework conditions must be met for you to consider health data sharing with research companies?
   7. Imagine that your health data would be automatically made available to research companies if you do not actively opt out. How does it feel?

*Timeframe: 20 minutes*

1. *How do you feel about the use of your health data by private companies?*
   1. Do you already have experience with this?
      - - If yes, how was your experience?
        - If no, what are possible reasons?
   2. What opportunities or advantages do you see in the use of your health data by private companies?
   3. What risks or disadvantages do you see in the use of your health data by private companies?
   4. What (type of) health data would you share with private companies?
   5. For which specific uses or purposes would you share your heath data with private companies?
   6. What framework conditions must be met for you to consider health data sharing with private companies?
   7. Imagine that your health data would be automatically made available to private companies if you do not actively opt out. How does it feel?

*(Timeframe: 20 minutes)*

**Concluding question**

*Are there any final important aspects that have not been addressed so far? Do you have anything else to add?*

*(Timeframe: 10-15 minutes)*
