# Supplementary material for: Perspectives on Health Data Sharing Among Patients With Somatic and Mental Health Diseases: Focus Group Study
Source: J Med Internet Res. 2026 Apr 13;28:e79990. doi: 10.2196/79990 (PMC13122138; doi:10.2196/79990)
Supplement: Multimedia Appendix 3 [file jmir_v28i1e79990_app3.docx]

| **Main category** | **Subcategory** | **Definition** | **Anchor example** | **Number / context of statements (PU, SU, G)** |
| --- | --- | --- | --- | --- |
| **Icebreaker question** | | **What is the first thing that comes to mind when you think about health data?** | „My lab results. I would like to have the same personal health information that the doctor has on the computer.“ FG1.7 | 14 |
| **I. Previous experience with data sharing** | | **Personal experiences with using and sharing of health data** |  | **38** |
|  | Health care professionals | Experiences in sharing health data with health care professionals | „I had cancer three years ago, and it was good that the doctor, from a completely different field knew right away what was going on [with my health data], and we took advantage of that.“ FG2.1 | 18 PU |
|  | Research and clinical studies | Experiences in sharing health data for research and clinical studies | „I haven’t taken part in any studies or anything like that.” FG1.2 | 8 SU |
|  | Employers and private companies | Experiences in sharing health data with employers and other private companies | „Well, I'm not entirely sure that the diagnostic code was on the form that the employer received.” FG2.5 | 5 SU |
|  | Health insurance companies | Experiences in sharing health data with health insurance companies | „I would like to add a comment here, though: adopting the electronic patient file, at least that’s quite complicated for me. It may be that every health insurance company handles this differently and mine is particularly complicated.“ FG1.7 | 4 PU |
|  | Medical education | Experiences in sharing health data for medical education | „I also work very closely [...] with the professors. I've often been to lectures or student meetings there [proving my medical history]. I think it's important that the students who are studying medicine or training to become nurses also get the chance to see how the patients are doing.“ FG2.4 | 3 SU |
| **II. Individual usefulness for medical care** | | **Personal benefits through improved individual health care to facilitate health data sharing** |  | **32** |
|  | Comprehensive documentation | Individual advantages through comprehensive documentation in the context of medical care | „If they had everything stored on the health card and the medical service, the doctors and everyone would had access to it straight away. That would make things much easier.“FG2.4 | 12 PU |
|  | Personal informedness | Advantages through overview, information and better organization for individuals | „I have to say, I have a folder at home that is now so thick [...] that would of course be an advantage for me if it were completely stored on the health card.“ FG2.4 | 6 PU |
|  | Emergency assistance | Health data sharing in case of emergency | „That would also be okay for me if it worked that way in an emergency: I'm admitted to the hospital, they take my health insurance card from my wallet and can access my data.“ FG1.4 | 5 PU |
|  | Simplification of processes | Optimization and simplification of processes and workflows in the context of medical care | „Because that would always simplify and accelerate many processes.” FG1.2 | 5 PU |
|  | Time saving | Time saving in doctor-patient contact | „I wouldn’t need to talk about it for long; they would have it directly in the app or on the [health insurance] card.” FG2.3 | 4 PU |
| **III. Public benefit** | | **Public benefits through improved medical care and possible cost savings for the population to facilitate health data sharing** |  | **28** |
|  | **Medical care** | Public benefits for medical care |  | **25** |
|  | Drug and product  development | Research potential for drug and product development | „So, I agree that companies do, of course, use such findings for the development of drugs and so on. That's fine.” FG1.3 | 12 SU |
|  | Gaining knowledge | Gaining knowledge in order to improve medical care | „A lot of insights can be gained from the health data collected.“ FG1.7 | 10 SU |
|  | Deriving preventive  measures | Derivation of preventive measures based on shared health data | „Or even [that] the health insurance company [use my health data], because they look at preventive measures, that’s okay too.” FG1.6 | 3 SU |
|  | Cost saving | Public benefits due to cost savings | „It's not the cost of medication that's the worst, but the cost of early retirement. That would be an incredibly great contribution, but that is not the case yet.” FG1.6 | 3 SU |
| **IV. Personal and privacy concerns** | | **Personal resistance and concerns about privacy violations as barriers for health data sharing** |  | **34** |
|  | Concerns about commercial interests | Concerns about and rejection of commercial health data use | „But it's not like anybody and everybody from Leipzig, Berlin, or wherever can come along and say, „Hey, we can make some money with research.“FG2.4 | 13 SU 2 G |
|  | Concerns being discriminated or stigmatized | Concerns about discrimination and stigmatization, and the possible consequences of these | „And not, […], health insurance company XY is currently considering that it no longer wants to accept expensive patients in the near future.” FG1.6 | 1 PU 5 SU 2 G |
|  | Personal uncertainties and skepticism | Personal insecurity and uncertainties, doubts and skepticism on digital health and health data sharing | „Because there is really too little development to be seen. At least not in the short time frame required for it to be achieved.“ FG1.7 | 1 PU 3 SU 3 G |
|  | Concerns being overstrained | Concerns about being overwhelmed or overstrained by health data sharing | „That can also develop into quite a big mental chaos, excuse me, but it can develop mental chaos quite honestly.” FG2.4“ | 2 PU 1 SU 1 G |
| **V. Data security concerns** | | **Data security concerns as barriers of health data sharing** |  | **34** |
|  | Concerns about data access | Concerns regarding data access eg, incorrect data recording by humans or AI, data access without consent, unauthorized data access, possible hacking | „So, basically, that’s the fear that everyone has. Who can access all the health data? And I'm still a little anxious about that sometimes.“ FG2.1 | 4 PU 6 SU 14 G |
|  | Concerns about data processing | Concerns regarding data processing eg, unauthorized data transfer, data processing without consent, possible re-identification | „But people would have absolutely no idea what happens to their data. They wouldn’t even know what studies their data is being used for.” FG2.2 | 2 PU 4 SU 4 G |
|  |  |  |  |  |
|  |  |  |  |  |
| **VI. Consent management preferences** | | **Individual preferences regarding design of the consent management model and its scope for the use and sharing of health data** |  | **128** |
|  | **Consent scope** | Preferred scope and applicability of the consent for health data sharing |  | **87** |
|  | **Type and format of**  **information** | Scope and applicability of consent in relation to the type and format of health data |  | **12** |
|  | Sensitive vs  nonsensitive data | Consent management preferences for the sharing of sensitive and non-sensitive health data | „There are infectious diseases, for example. I wouldn’t know whether I would authorize everyone to see them.” FG1.5 | 6 PU |
|  | Anonymized and  pseudonymized data | Consent management preferences for the sharing of anonymized and pseudonymized health data | „But really just the anonymized collection of the data. And I would agree to that without any reservations.” FG1.7 | 4 SU |
|  | Emergency data | Consent management preferences for the sharing of emergency data | „I think that doctors, who know it, need to differentiate: what health information is vital if I have an accident and cannot speak and do not have the health [insurance] card with me? So, what is he allowed to be told at the hospital?“ FG2.6 | 2 PU |
|  | **Recipient and purpose** | Scope and applicability of consent in relation to recipient and purpose of health data sharing |  | **75** |
|  | Health care  professionals | Consent management preferences for health data sharing with health care professionals | „In principle, I feel the same way about selection, as I would intuitively say that not every doctor should be able to see everything.” FG1.4 | 29 PU |
|  | Research  and pharmaceuticals | Consent management preferences for the health data sharing with pharmaceutical research companies | „And yes, the data is of course also important for the technical university here and everything. And I'm definitely not against the data being used for research.“ FG2.4 | 32 SU |
|  | Private companies | Consent management preferences for the health data sharing with private companies | „For me, I wouldn’t make that much of a distinction between medical device manufacturers and other manufacturers.” FG1.2 | 14 SU |
|  | **Consent model** | Preferred model and design of consent for health data sharing |  | **41** |
|  | Opt-in | Active approval and request for permission to the use and sharing of health data; explicit and flexible consent | „For data that cannot be anonymized, such as patient files, patient doctor reports and such, I would actually prefer the opt-in option authorized via my health [insurance] card.“ FG1.4 | 4 PU 11 SU 4 G |
|  | Opt-out | Basic, default permission and active, explicit rejection of the use and sharing of health data | „So, with this option, I would be able to deselect something, i.e. reject someone access. But in general, if I don't say anything, I want to allow access.“ FG1.7 | 11 PU 5 SU 6 G |
| **VII. Technical safety measures** | | **Technical safety measures for data storage, access and transfer to facilitate health data sharing** |  | **37** |
|  | Anonymization and pseudonymization | Anonymization and pseudonymization procedures in the context of data storage and transfer | „Well, it probably won't be anonymized, but pseudonymized, I assume, because somehow you have to ensure that certain data isn't included multiple times and things like that, which wouldn't be possible with anonymization.” FG1.4 | 16 SU 6 G |
|  |  |  |  |  |
|  | Central data repository | Central data repository of digital health data | „Well, what would be nice, but that would be more like the icing on the cake, is, if everything would be stored in a central system and where it is recorded who has access to it anyway.” FG1.4 | 2 PU 3 SU 3 G |
|  | Data encryption | Data encryption techniques in the context of health data access | „Exactly, and then the rest of the data, which can perhaps also be encrypted in parts. And when I go to the doctor, the doctor shows me what he wants to open and I enter a PIN.” FG2.6 | 5 PU 2 G |
| **VIII. Legal and ethical framework conditions** | | **Legal and ethical framework conditions to facilitate health data sharing** |  | **35** |
|  | Data protection | Required data protection regulations | „But that would have to be protected by a proper PIN so that only doctors, and I personally can access the [health insurance] card.” FG2.4 | 2 PU 3 SU 6 G |
|  | Standardization and regulation | Standardization and regulation of health data sharing | „And such regulations and procedures are needed to get things off the ground.” FG1.1 | 3 PU 7 G |
|  | Ethical justificability | Ethical justificability for the use of shared health data | „I think that would be a general rule. And as before, they would still have to specify at the beginning what they want to research. This ethical component would need to be clarified.” FG1.6 | 5 SU 2 G |
|  | Monitoring and authorization | Required monitoring and authorization measures | „The question is who will monitor all the clever ideas that have been come up with. As he said, there needs to be a committee.“ FG1.1 | 3 SU 4 G |
| **IX. Informational self-determination** | | **Factors of informational self-determination to facilitate health data sharing** |  | **33** |
|  | Transparency | Transparency regarding technical mechanisms, health data use, and outcomes of health data sharing | „If it were a manageable list, then it would actually be quite satisfying to see what happens with the data.” FG1.4 | 1 PU 9 SU 8 G |
|  | Autonomy and control | Self-determined and independent control, freedom of choice, and sovereignty | ”I want to decide for myself which doctor sees what.“ FG2.3 | 2 PU 4 SU 2 G |
|  | Trust | Trust and trust-building measures | „I would share my data. Well, for solid research, or what does solid research mean? That I trust.“ FG2.6 | 3 SU 4 G |
| **X. Social involvement and influence** | | **Involvement and influence of the personal social environment** |  | **9** |
|  | Family and peers | Involvement and influence of family members and friends in the context of health data sharing | „I think it's very important that relatives who are in possession of a living will or similar document are able to share their health data in case of a medical emergency.“ FG2.4 | 4 PU 1 G |
|  | Health care professionals | Involvement and influence of health care professionals in the context of health data sharing | „That's how it was explained to me. In principle, I am responsible for this [patient] file. So I can say that this doctor can see certain things and that doctor cannot see certain things.” FG2.3 | 3 PU 1 G |
| The codes were always assigned at the lowest level of the relevant subcategory.  Primary Use (PU) = statement relating to primary use (eg, medical treatment); Secondary Use (SU) = statement relating to secondary use (eg, medical research); General (G) = general statement, cannot be assigned to either PU or SU | | | | |
