# Supplementary material for: Perspectives on Health Data Sharing Among Patients With Somatic and Mental Health Diseases: Focus Group Study
Source: J Med Internet Res. 2026 Apr 13;28:e79990. doi: 10.2196/79990 (PMC13122138; doi:10.2196/79990)
Supplement: Multimedia Appendix 4 [file jmir_v28i1e79990_app4.docx]

| **Variable** | **FG1** | **FG2** |
| --- | --- | --- |
| *Age (years)* | | |
| 18 – 40 | 2 | 3 |
| 41 – 64 | 2 | 2 |
| ≥ 65 | 3 | 1 |
| *Gender* | | |
| Female | 3 | 3 |
| Male | 4 | 3 |
| *Education* | | |
| Intermediate secondary school | 1 | 3 |
| University entrance qualification | 6 | 3 |
| *Vocational education and training* | | |
| Completed apprenticeship | 2 | 5 |
| University degree | 4* | 1 |
| Doctoral degree | 1* |  |
| *Current employment* | | |
| In apprenticeship | 1 |  |
| Marginal or irregular employment | 1 | 2 |
| Part-time employment |  | 2 |
| Full-time employment | 2 |  |
| (Early) retirement | 3 |  |
| Unemployment |  | 1 |
| Unable to work |  | 1 |
| *Household income (per month, in €)* | | |
| < 1.000 – 1.500 | 1 | 4 |
| 1.501 – 2.000 | 1 |  |
| 2.001 – 3.000 |  |  |
| 3.001 – 3.500 | 3 | 1 |
| 3.501 – 4.000 |  |  |
| 4.001 – 5.000 | 2 |  |
| No information |  | 1 |
| *Residential area* | | |
| Rural community (population < 5.000) | 1 |  |
| Medium-sized city (population 20.000 – < 100.000) | 2 | 1 |
| Large city (> 100.000) | 4 | 5 |
| *Distance to nearest large city* |  |  |
| No distance | 1 | 3 |
| < 10km | 4 | 2 |
| > 10 – < 25km | 1 | 1 |
| ≥ 25km | 1 |  |

* categorized according to the highest degree
