# Supplementary material for: Perspectives on Health Data Sharing Among Patients With Somatic and Mental Health Diseases: Focus Group Study
Source: J Med Internet Res. 2026 Apr 13;28:e79990. doi: 10.2196/79990 (PMC13122138; doi:10.2196/79990)
Supplement: Multimedia Appendix 5 [file jmir_v28i1e79990_app5.docx]

| **FG and participant** | **Technology acceptance** | **Technology competence beliefs** | **Technology control beliefs** | **Total score** |
| --- | --- | --- | --- | --- |
| *FG1* | | | | |
| F1.1 | 16 | 15 | 16 | 47 |
| F1.2 | 13 | 18 | 16 | 47 |
| F1.3 | 18 | 20 | 19 | *57* |
| F1.4 | 18 | 20 | 14 | 52 |
| F1.5 | 12 | 13 | 16 | *41* |
| F1.6 | 16 | 20 | 18 | 54 |
| F1.7 | 14 | 20 | 20 | 54 |
| *FG2* | | | | |
| F2.1 | 14 | 14 | 12 | 40 |
| F2.2 | 12 | 15 | 13 | 40 |
| F2.3 | 12 | 17 | 18 | 47 |
| F2.4 | 16 | 20 | 14 | 50 |
| F2.5 | 12 | 14 | 12 | *38* |
| F2.6 | 16 | 20 | 20 | *56* |
